# A Comparative Study between Screen-Printed and Solid-Contact Electrodes for the Stability-Indicating Determination of Bromazepam

**DOI:** 10.3390/molecules27217616

**Published:** 2022-11-06

**Authors:** Sherif A. Abdel-Gawad, Ali Altharawi

**Affiliations:** 1 Department of Pharmaceutical Chemistry, College of Pharmacy, Prince Sattam Bin Abdulaziz University, Al Kharj 11942, Saudi Arabia; 2 Analytical Chemistry Department, Faculty of Pharmacy, Cairo University, Cairo ET-11562, Egypt

**Keywords:** stability-indicating, screen-printed electrode, solid-contact electrode, PVC-based membrane electrode

## Abstract

Stability-indicating methods are awesome tools to ensure the safety and efficacy of active pharmaceutical ingredients (APIs). An accurate comparative study involving the use of potentiometric sensors for the determination of bromazepam (BRZ) in the presence of its main product of degradation and impurity was performed by the fabrication of two membrane electrodes. A screen-printed electrode (SPE) and a solid-contact glassy carbon electrode (SCE) were fabricated and their performance optimized. The fabricated sensors showed a linear electrochemical response in the concentration range 1.0 × 10^−6^ M to 1.0 × 10^−2^ M. The electrodes exhibited Nernstian slopes of 59.70 mV/decade and 58.10 mV/decade for the BRZ-SPE and BRZ-SCE membrane electrodes, respectively. The electrochemical performance was greatly affected by the medium pH. They showed an almost ideal electrochemical performance between pH 3.0 and pH 6.0. The fabricated membranes were applied successfully for the quantification of BRZ in the presence of up to 90% of its degradation product. Moreover, a successful application of the fabricated electrodes was performed for the sensitive and selective quantification of BRZ in its tablet form without any pretreatment procedure.

## 1. Introduction

Stability-indicating assays can be considered an awesome tool to assess the stability, efficacy, and safety of the active pharmaceutical agent (APA). They can detect and quantify the intact APA in the presence of its degradation product. The occurrence of a degradation product or an impurity with the APA can greatly affect its toxicological, chemical, and pharmacological actions, which, in turn, affect the safety and quality of the final pharmaceutical product [1]. Bromazepam (BRZ) as with all 1,4-benzodiazepines has hypnotic, muscle relaxant, and sedative effects [2]. These actions are attributed to the stimulation of the gamma-aminobutyric acid (GABA) receptors in the brain [3]. BRZ is widely used for handling insomnia and anxiety disorders [4,5,6]. 

British Pharmacopeia (BP) reports a non-aqueous method for the analysis of BRZ in bulk form. It depends on the use of HClO_4_ as a titrant [7]. Many analytical techniques have been reported in the literature for the assay of BRZ either alone or in conjunction with other drugs in dosage form and/or body fluids. These techniques include ultraviolet (UV)–visible spectroscopy [8,9,10,11,12], fluorimetry [8,13], liquid chromatography (LC) [14,15,16,17,18,19,20,21,22,23,24,25,26], gas chromatography connected to mass spectrometry (GS/MS) [27,28,29], and capillary electrophoresis (CE) [30,31]. Potentiometric membrane sensors, either inner liquid [32,33,34] or solid-contact electrodes [35], were used for the determination of BRZ in its tablet form, biological fluids, and wastewater effluents.

**Figure 1:**
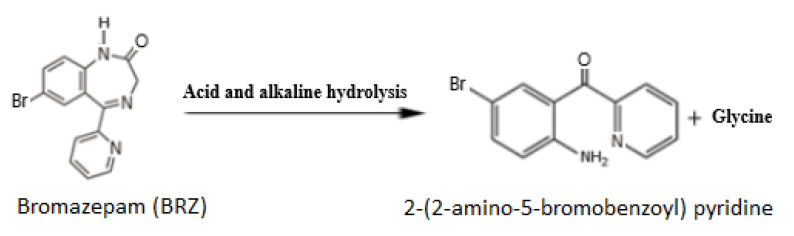
BRZ degradation pattern via acid and alkaline hydrolysis [36].

Regarding the chemical stability of BRZ, it exhibits rapid degradation following the reaction indicated in Figure 1 [36]. The main degradation product is 2-(2-amino- 5-bromobenzoyl) pyridine (ABBP), which is also considered a potential impurity commonly present in BRZ bulk form, as reported by the BP [7]. Moreover, the main metabolic pathway of BRZ involves the formation of two metabolites, which are ABBP and 3-hydroxybromazepam [37,38]. Many analytical methods were reported as stability-indicating methods for BRZ. These methods applied spectroscopic [36,39] and liquid chromatographic [40,41] techniques for the selective determination of BRZ. 

Ion-selective electrodes (ISEs) are considered as a tool used for the reliable and accurate determination of a lot of different analytes as they are economic, time saving, and non-destructive [42,43,44,45]. This work aims to introduce two ISEs for the sensitive, selective, and accurate stability-indicating quantification of BRZ in the presence of its main degradation product. The proposed method has advantages over the previously published stability-indicating methods. It is simpler, more economic, and does not need a special instrument assembly or complicated data processing. Moreover, the cited electrochemical method does not need a special sample pretreatment procedure. After an extensive literature survey, no electrochemical methods have been published for the quantification of BRZ in the presence of its main degradation product.

**Figure 2:**
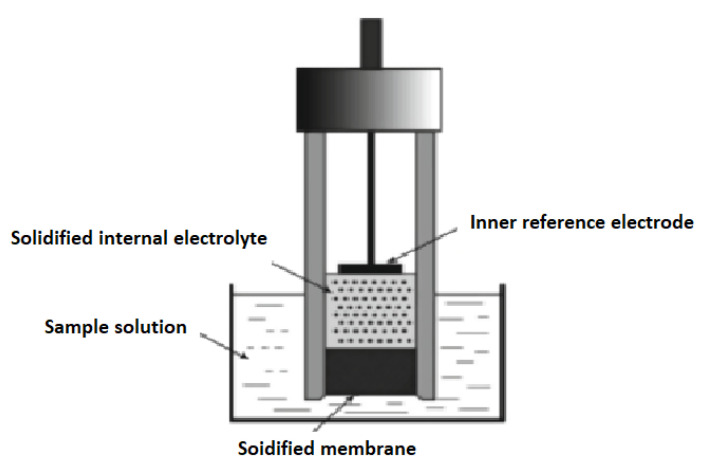
Schematic diagram for the solid-contact electrode.

Solid-contact electrodes (SCEs) have recently become a popular electrochemical technique to overcome the problems of liquid contact electrodes, where they eliminate the use of the inner filling solution and replace it with a solid-state junction among the membrane and the metal electrode [46] (Figure 2).

In the last few years, screen-printed electrodes (SPEs) have become an important tool in the electrochemical application, especially in the bio-sensing and environmental analysis era [47]. The simplicity with which SPEs’ surfaces can be modified in a variety of ways is a source of enormous creativity. Because of their fast response, design flexibility, small sample size, excellent sensitivity, and lack of pretreatment processes, SPEs are considered one of the best electrodes for in situ analysis [48,49].

In view of the mentioned merits of the SPE and SCE, two distinct BRZ membrane sensors, i.e., an SPE and an SCE, are fabricated. A comparative study is conducted between the fabricated sensors regarding their working parameters. Furthermore, the fabricated sensors are applied as a stability-indicating approach for the sensitive and selective determination of BRZ in the presence of its main degradation product, either in bulk or dosage forms. It is worth mentioning that ABBP is also the main metabolite of the studied drug.

## 2. Results 

The fabrication and validation of effective membrane sensors, which can be effectively applied for the sensitive and accurate quantification of APAs in the presence of their degradation products, is an awesome advantage. In this work, potentiometric membrane sensors (SPE and SCE) are fabricated, validated, and applied as an economic tool having the merits of minimal sample preprocessing and high accuracy for the stability-indicating determination of BRZ in the presence of its main degradation product, which at the same time, is considered as its major metabolite and main impurity. 

### 2.1. Manufactured Membranes’ Calibration and Performance Evaluation

To validate the efficiency of the fabricated electrodes, the electrochemical performance was optimized according to the International Union of Pure and Applied Chemistry (IUPAC) standards [50].

**Figure 3:**
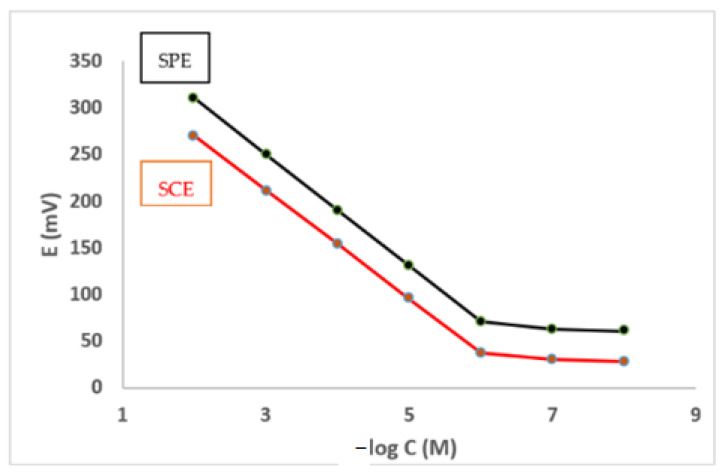
Calibration graphs for the BRZ electrodes at 25 °C.

The performance assessment data for the fabricated electrodes are shown in Table 1. A nearly optimal Nernstian slope was achieved in the concentration range 1.0 × 10^−6^ M and 1.0 × 10^−2^ M for the BRZ solutions, for both fabricated electrodes. The slopes of the SPE and SCE were 59.70 ± 0.40 mV/decade and 58.10 ± 0.60 mV/decade, respectively, at pH 3.0–6.0 (Table 1). Figure 3 shows typical calibration graphs at 25 °C.

Trials with three different plasticizers of various polarities were conducted to determine the impact of plasticizer type (o-NPOE, DOP, and DBS) on the electrochemical behavior of the fabricated sensors. The optimum behavior was achieved with o-NPOE, whose manufactured sensors had slopes that were closer to the ideal Nernstian values, indicating more successful ion exchange through the fabricated electrode. On the other hand, the membranes fabricated using DOP showed slopes of 50.70 ± 0.60 mV/decade (n = 5) and 49.90 ± 0.60 mV/decade (n = 5) for the SPE and SCE, respectively (Table S1 and Figure S1), and those manufactured using DBS as a plasticizer exhibited slopes of 51.80 ± 0.50 mV/decade (n = 5) and 50.50 ± 0.50 mV/decade (n = 5) for the SPE and SCE, respectively (Table S2 and Figure S2).

**Figure 4:**
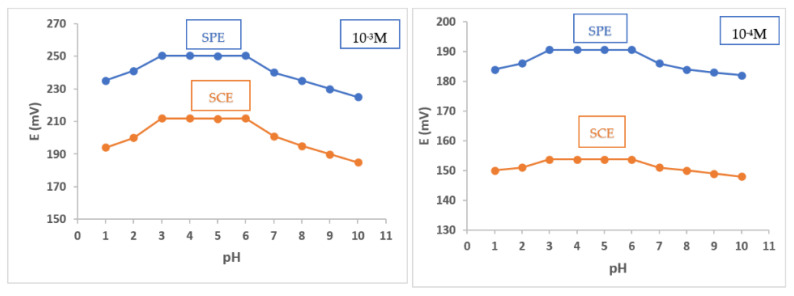
pH effect on the potential response of the fabricated electrodes using 1.0 × 10^−3^ M and 1.0 × 10^−4^ M BRZ standard solutions.

The pH of the medium has an impact on the functioning of the manufactured sensors. It was tested in the pH range of 1.0 to 10.0. The various pH values were achieved by the dropwise addition of 1.0 × 10^−2^ M HCl and 1.0 × 10^−2^ M NaOH on 1.0 × 10^−3^ M and 1.0 × 10^−4^ M BRZ standard solutions (Table S3). The best electrochemical response was observed in the pH range of 3.0–6.0. (Figure 4). 

**Figure 5:**
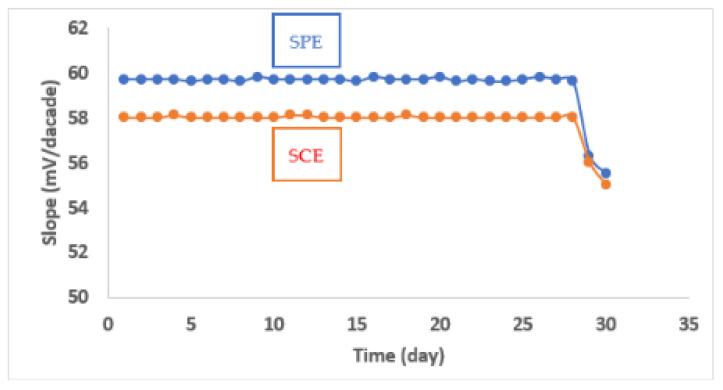
Stability of the fabricated membrane sensors at 25 °C.

The manufactured electrodes’ lifetimes were 28 days for the SPE and SCE. The acquired potentials at 25 °C were stable in this time period, as shown in Figure 5. When using the SPE and SCE for BRZ standard solutions in its corresponding concentration linearity range, the response times were 10–20 S. When the sensors were used for potential measurement in concentrations below 1.0 × 10^−6^ M, however, the response time increased to be 20–30 s. 

Assay accuracy was checked via the application of the fabricated sensors to analyze three BRZ concentrations (1.0 × 10^−2^ M, 1.0 × 10^−3^ M, and 1.0 × 10^−4^ M), and each concentration was measured twice, giving acceptable results (Table S4). Precision of the proposed method was evaluated by the accurate analysis of three BRZ concentrations (1.0 × 10^−2^ M, 1.0 × 10^−3^ M, and 1.0 × 10^−4^ M), and each concentration was measured twice, either intra-day or inter-day to confirm method repeatability. At the same time, evaluation of the method robustness was carried out upon performing a slight pH variation (6.2) during the application of the fabricated sensors for the analysis of three BRZ concentrations (1.0 × 10^−2^ M, 1.0 × 10^−3^ M, and 1.0 × 10^−4^ M), and each sample was measured twice (Table S5). Method ruggedness was tested via the accurate analysis of three BRZ concentrations, and each concentration was measured twice, by different sensor assemblies, in order to confirm method reproducibility (Table S6). Table 1 shows the electrochemical response characteristics of the fabricated electrodes.

The fabricated membranes’ selectivity was assessed by the separate solution method (SSM) [51]. Table 2 shows the potentiometric selectivity coefficients (PSCs) of the SPE and SCE electrodes in the presence of different interferents (various inorganic ions, BRZ degradation product (ABBP), and structurally related benzodiazepines). The results declared excellent selectivity of the developed sensors for the studied drug.

### 2.2. Determination of BRZ in Presence of Its Main Degradation Product

The cited method was tested for its selectivity to determine BRZ in the presence of different ratios of its main degradation product (10–90%). This mission was performed by applying the fabricated sensors to analyze different laboratory-prepared mixtures having variable ratios of the intact BRZ and its main degradation product. The assay results are given in Table 3, which declares the capability of the quantification of BRZ in the presence of up to 90% of its main degradation product.

### 2.3. Determination of BRZ in Pharmaceutical Preparation

The cited method was applied for the analysis of Lexotanil^®^ tablets, and the results are introduced in Table 4. The obtained results showed that the fabricated electrodes can be successfully used for the assay of BRZ in its tablet form without the interference of any additives or excipients. 

### 2.4. Statistical Analysis with a Reported Method 

Table 5 shows a statistical study of the results obtained for the assay of BRZ in pure form using the cited method versus results obtained using a published HPLC technique [23]. The calculated t and F values are lower than the tabulated ones, indicating that there is no substantial difference in accuracy and precision between the two approaches.

## 3. Discussion

The quantification of intact drugs in the presence of their degradation products is a crucial task in order to guarantee their safety, efficacy, and quality. The application of potentiometric ISEs in the stability-indicating determination of BRZ is regarded as an important analytical tool. 

In our previously published article [34], liquid-contact electrodes were fabricated using either sodium tetra-phenyl borate or phosphotungstic acid as ion-pairing agents for the detection and quantification of the studied drug in industrial wastewater effluents. In the present work, other types of potential membrane sensors (SPE and SCE) were fabricated to perform another task, which was the stability-indicating determination of BRZ in the presence of its main degradation product. The fabricated electrodes in the present work have many advantages over the fabricated electrodes in the previous work. They avoid the tedious refilling process needed in the liquid-contact electrodes. Moreover, the SPE and SCE are characterized by a wider linearity range and longer lifespan. 

First of all, the working conditions for the fabricated electrodes were optimized according to the IUPAC recommendations [50]. 

There is no doubt that the selection of a suitable plasticizer plays an important role in the optimum electrochemical behavior of the fabricated sensors. o-NPOE was used as a plasticizer in the fabrication of a lot of ISEs to facilitate the ion transport process across the membrane [52]. It was tried in this work together with another two plasticizers (DBS and DOP) to select the most suitable one. The plasticizer that gave the optimum performance of the two fabricated electrodes was o-NPOE. This may be attributed to the similar polarities of BRZ and o-NPOE that ensure the most successful ion exchange across the fabricated sensors. 

The medium pH plays an important role in obtaining the optimum electrode performance, which is ensured in a range of pH from 3.0 to 6.0. A noticed deviation from the standard curves’ linearity was observed at pH levels below three, which can be attributed to the interference encountered by the elevated hydronium ions [32,33,34]. On the other hand, at pH values above six, the loss of calibration curve linearity can be attributed to the decrease in BRZ solubility, leading to a marked decrease in electrode potentials [53].

Regarding the lifetime and long-term stability, the manufactured electrodes exhibited excellent potential stability for up to 28 days, which is a comparable value with the reported BRZ potentiometric ISEs (21 days–28 days), as declared in Table 1. After this time the stability of the manufactured sensors was significantly reduced. This might be due to the ion-pair leakage from the manufactured sensors and leaching of the membrane components. 

The fabricated electrodes were compared to those in published methods comprising comparable potentiometric ISEs (Table 1). The proposed membranes have a wider linearity range with comparable sensitivity, response time, and stability. The fabricated sensors overcome the problem of inner liquid refilling faced in the liquid-contact membranes fabricated in the published methods [32,33,34]. The cited potentiometric membrane sensors [32,33,34,35] were not all used as stability-indicating methods of analysis, which can be considered a marked advantage for the proposed method. Moreover, the proposed method applied the SPE as a stability-indicating tool, which was not found in the other published methods.

Method accuracy was evaluated by analyzing different BRZ concentrations using the fabricated sensors giving excellent recoveries. Precision, either intra-day or inter-day, was checked by analyzing different BRZ concentrations, either on the same day or on three successive days, giving good recoveries and ensuring method repeatability. Method ruggedness was evaluated by analyzing different BRZ samples using different sensor assemblies to evaluate that the proposed method can withstand the inter-laboratory variations. On the other hand, method robustness was checked to evaluate the capability of the method to tolerate deliberate changes in the experimental conditions (e.g., pH). 

Membrane selectivity is well validated using the SSM in the presence of different interferents, including different inorganic ions (Na^+^, K^+^, and NH_4_^+^), BRZ degradation product (ABBP), and structurally-related benzodiazepines (diazepam and clonazepam), giving excellent values of the PSCs ensuring the sensors’ specificity to the studied drug.

The fabricated electrodes are successfully applied for the determination of BRZ in the presence of up to 90% of its degradation product, which makes the proposed method a very effective tool in stability studies. Moreover, the membranes are applied for the determination of BRZ in its tablet form without the need for any pretreatment or separation steps, which gives this method an advantage over the published stability-indicating methods for the studied drug. 

## 4. Materials and Methods

### 4.1. Instrumentation

The potentiometric measurements were conducted using a Jenway potentiometer of model 3510 supplied from Jenway (London, United Kingdom). It was supplied with a reference electrode (Ag^0^/AgCl). A Jenway pH meter (London, United Kingdom) was utilized for pH measurement all over the study. The SCE was set by glassy carbon rod of 3.0 mm diameter. They were purchased from CH Instruments (Austin, TX, USA). The SPEs were prepared using screen-printed solid support sheets, their dimensions were 50.0 mm × 13.0 mm. They were supplied from CH Instruments (Austin, TX, USA). The prepared electrodes were connected to a saturated Ag/AgCl reference electrode. 

### 4.2. Chemicals, Reagents, and Dosage Form

The BRZ pure substance was obtained from Hoffmann-La Roche (Basel, Switzerland). It was of 99.92% purity. ABBP was purchased from Molbase chemicals (Shanghai, China). Its purity was certified to be 99.83%. Lexotanil^®^ tablets labeled to contain 3 mg BRZ per tablet were purchased. Sodium tetraphenylborate (TPB), AgNO_3_, sodium hydroxide (NaOH), HCl, and tetrahydrofuran (THF) were purchased from Sigma-Aldrich (Darmstadt, Germany). Dibutyl sebacate (DBS), dioctyl phthalate (DOP), polyvinyl chloride (PVC), and o-nitrophenyl octyl ether (o-NPOE) were supplied from Prolabo (Nantes, France). The water used in this work is bi-distilled.

### 4.3. Standard Solutions

#### 4.3.1. BRZ Standard Solutions

Preparation of BRZ stock standard solution (1.0 × 10^−2^ M) was conducted by dissolving 0.32 gm BRZ into 30.0 mL 1.0 × 10^−2^ M HCl. Complete drug dissolution was performed via the dropwise addition of 2.0 M hydrochloric acid solution with continuous stirring. Distilled water was used to adjust the volume of the resulting solution to 100.0 mL in a volumetric flask. The prepared solution was used to prepare BRZ working solutions (1.0 × 10^−3^ M to 1.0 × 10^−8^ M) via dilution using distilled water.

#### 4.3.2. Preparation of Sodium Tetraphenylborate (Na-TPB) Solution 

It was carried out by the accurate weighing of 0.68 g Na-TPB and dissolving in 100.0 mL distilled water to reach the concentration of 1.0 × 10^−2^ M. Standardization of the prepared solution was performed through the application of potentiometric titration against AgNO_3_ of the same molarity.

### 4.4. Procedures

#### 4.4.1. Preparation of the BRZ-SCE

o-NPOE (0.40 g), TPB (0.01 gm), and PVC (0.19 gm) were blended in a Petri dish. The mixed powders were dissolved using six mL THF. Direct application of the prepared solution to a glassy carbon electrode was performed by dipping the top of the glassy carbon electrode in the prepared solution mixture three times, each time for three seconds. The electrode was left overnight to allow complete evaporation of the solvent at room temperature.

#### 4.4.2. Preparation of the BRZ-SPE

The electrode was made via drop-casting of the previously attained liquid mixture (o-NPOE, TPB, and PVC in THF) over the carbon working electrode of the screen-printed solid support. The assembly was left to air dry for 24 h.

#### 4.4.3. Sensors’ Conditioning and Calibration 

Conditioning of the prepared electrodes was performed via soaking in 1.0 × 10^−2^ M BRZ solution for one day. The prepared electrode was stored in the same BRZ solution when not used. On the other hand, the calibration process was performed by dipping the conditioned electrodes in BRZ standard solutions (1.0 × 10^−8^ M, 1.0 × 10^−7^ M, 1.0 × 10^−6^ M, 1.0 × 10^−5^ M, 1.0 × 10^−4^ M, 1.0 × 10^−3^ M, and 1.0 × 10^−2^ M) in conjunction with saturated Ag/AgCl reference electrode. The potential readings were used to plot the calibration graphs with the corresponding BRZ concentrations.

#### 4.4.4. Sensors’ Optimization and Validation

Optimum membrane performance was attained through the studying and optimization of the various parameters affecting its works. The electrochemical performance of the fabricated sensors was validated along with the IUPAC regulations [50].

The plasticizer characters may strongly affect the fabricated electrode performance. This issue was checked by using different plasticizers with variable polarities (DBS, DOP, and o-NPOE). This step was conducted to select the plasticizer that gives the optimum electrochemical performance. 

BRZ standard solutions (1.0 × 10^−3^ M and 1.0 × 10^−4^ M) were used in studying the pH effect on the performance of the fabricated sensors. The pH values were changed by the dropwise addition of 1.0 × 10^−2^ M NaOH or HCl. The obtained potential values were plotted against the pH values to obtain the optimum pH range in which an optimum performance was attained for the fabricated sensors.

The lifetime and long-term stability of the prepared sensors were evaluated by repeating the sensors’ calibration for one month. The potential was traced for a BRZ concentration range 1.0 × 10^−2^ M–1.0 × 10^−6^ M every day. The potential slope was measured for each sensor and compared with that obtained during the first time calibration. 

The accuracy of the method was evaluated by using the fabricated sensors for analyzing three BRZ samples (1.0 × 10^−2^ M, 1.0 × 10^−3^ M, and 1.0 × 10^−4^ M), and each sample was measured twice. Precision (repeatability) was checked by analyzing three different BRZ concentrations (1.0 × 10^−2^ M, 1.0 × 10^−3^ M, and 1.0 × 10^−4^ M) either on the same day (intra-day) or on three successive days (inter-day), and each sample was measured twice. Ruggedness of the adopted method was evaluated by adopting the proposed method using different sensor assemblies (Hanna digital ion analyzer) for analyzing three different BRZ concentrations (1.0 × 10^−2^ M, 1.0 × 10^−3^ M, and 1.0 × 10^−4^ M), and each sample was measured twice. Additionally, evaluation of the method robustness was carried out upon performing slight pH variation (6.2) during the application of the fabricated sensors for the analysis of three BRZ concentrations (1.0 × 10^−2^ M, 1.0 × 10^−3^ M, and 1.0 × 10^−4^ M), and each sample was measured twice. 

Membrane selectivity was checked using the SSM [51]. The potential responses of the fabricated electrodes in the presence of different interferents, including different inorganic ions (Na^+^, K^+^, and NH_4_^+^), BRZ degradation product (ABBP), and structurally-related benzodiazepines (diazepam and clonazepam), were recorded and used for the calculation of the PSC for each interferent, where the potentials were measured for 1.0 × 10^−3^ M BRZ standard solution and then for 1.0 × 10^−3^ M aqueous interferent solution, separately, then the PSCs were calculated using the following equation:

PSC = (E_1_ − E_2_)/S,

where “E_1_” is the potential measured in 1.0 × 10^−3^ M primary ion (BRZ) solution, “E_2_” is the potential measured in 1.0 × 10^−3^ M interferent solution, and “S” represents the slope of the investigated sensor (mV/concentration decade).

### 4.5. Application 

#### 4.5.1. Quantification of BRZ in Presence of Its Degradation Product

Complementary volumes of BRZ and ABBP standard solutions were mixed to prepare different laboratory-prepared mixtures containing from 90% to 10% intact BRZ and from 10% to 90% ABBP. Careful adjustment of the prepared mixtures’ pH to a value in the range of 3.0–6.0 was carried out. The potentials of the prepared mixtures were measured with the help of the fabricated sensors in conjunction with saturated Ag/AgCl reference electrode. The concentration of BRZ was calculated with the aid of the plotted calibration graphs. 

#### 4.5.2. Determination of BRZ in Pharmaceutical Preparation

Weighing and crushing of twenty Lexotanil^®^ tablets from two different batches were performed, then tablet powder equivalent to 0.03 g BRZ was transferred to 100-mL volumetric flask. Thirty mL 1.0 × 10^−2^ M HCl were added. Continuous stirring was carried out for 2 min and the pH was adjusted to a value in the range of 3.0–6.0. Distilled water was used to adjust the volume of the prepared solution to 100.0 mL. The potential was measured using the fabricated sensors, and the BRZ concentration was calculated with the help of the plotted calibration graphs.

## 5. Conclusions

A study was conducted to compare two commercially unavailable BRZ ion-sensitive electrodes (SPE and SCE), that are characterized by their high selectivity and sensitivity for the studied drug in the presence of its main degradation product. The electrochemical behavior of the fabricated sensors was optimized according to the IUPAC recommendations. The fabricated sensors are well applied for the sensitive and selective quantification of BRZ in the presence of up to 90% of its main degradation product. The fabricated electrodes are successfully applied for the selective and sensitive quantification of the studied drug in its dosage form without the tedious pretreatment or separation steps. The SPE and SCE can be considered good choices for in situ BRZ quantification in quality control laboratories and those applying stability studies.

## Figures and Tables

**Table 1:** Electrochemical response characteristics of the fabricated electrodes.

Parameter	BRZ-SPE	BRZ-SCE	Published Method [32]	Published Method [33]	Published Method [34]	Published Method [35]
Slope (mV decade^−1^) *	59.70 ± 0.40	58.10 ± 0.60	52.00 ± 0.10	50.00–65.00	57.00 ± 0.22	50.00–65.00
Response time (S)	10–20	10–20	>20	-	10–20	
Working pH range	3.0–6.0	3.0–6.0	3.0	3.0–7.0	3.0–6.0	4.0–7.0
Concentration range (M)	1.0 × 10^−6^–1.0 × 10^−2^	1.0 × 10^−6^–1.0 × 10^−2^	1.0 × 10^−4^–1.0 × 10^−2^	1.0 × 10^−6^–1.0 × 10^−4^	1.0 × 10^−6^–1.0 × 10^−3^	-
Stability (days)	28	28	28	28	21	28
Accuracy (Mean ^a^ ± SD)	100.07 ± 1.06	100.42 ± 0.93	NA	-	99.71 ± 0.84	-
Intra-day precision (Mean ^a^ ± SD)	101.55 ± 1.21	101.11 ± 1.45	NA	-	-	-
Inter-day precision (Mean ^a^ ± SD)	102.13 ± 1.45	101.79 ± 1.69	NA	-	-	-
Limit of detection (M)	0.8 × 10^−6^	0.8 × 10^−6^	30.0 × 10^−6^	-	0.8 × 10^−6^	-
Ruggedness ^†^	99.51 * ± 1.28	98.41 * ± 1.66	NA	-	102.96 * ± 1.97	-
Robustness ^Ψ^	101.14 * ± 1.13	101.64 * ± 0.84	NA	-	99.36 * ± 0.59	-

* Average results of five determinations at 25 °C. ^a^ Average of three concentrations. ^†^ Comparing the results with those obtained by different sensor assemblies using Hanna digital ion analyzer. ^Ψ^ Carried out by measuring different known BRZ concentrations upon slight pH change.

**Table 2:** Potentiometric selectivity coefficients of the proposed bromazepam-selective sensors by the separate solution method using 1.0 × 10^−3^ M solutions of the interferent and BRZ at 25 °C.

Interferent	BRZ-SPE *	BRZ-SCE *
Na^+^	2.50 × 10^−3^	2.30 × 10^−3^
K^+^	3.60 × 10^−3^	3.50 × 10^−3^
NH_4_^+^	1.30 × 10^−3^	1.50 × 10^−3^
BRZ degradation product (ABBP)	4.10 × 10^−3^	4.20 × 10^−3^
Diazepam	4.90 × 10^−3^	5.10 × 10^−3^
Clonazepam	4.40 × 10^−3^	4.60 × 10^−3^

* Average of five measurements.

**Table 3:** Results obtained for the analysis of laboratory-prepared mixtures containing different ratios of intact bromazepam and its main degradation product by the proposed method at 25 °C.

Intact BRZ	Degradation Product (ABBP)	BRZ-SPE Recovery% ± S.D. *	BRZ-SCE Recovery% ± S.D. *
90%	10%		
9.0 × 10^−3^ M	1.0 × 10^−3^ M	100.89 ± 0.53	102.34 ± 0.93
70%	30%		
7.0 × 10^−4^ M	3.0 × 10^−4^ M	101.55 ± 0.73	101.46 ± 0.78
50%	50%		
5.0 × 10^−5^ M	5.0 × 10^−5^ M	100.93 ± 0.68	101.24 ± 0.55
30%	70%		
3.0 × 10^−6^ M	7.0 × 10^−6^ M	101.59 ± 0.56	102.43 ± 0.76
20%	80%		
2.0 × 10^−4^ M	8.0 × 10^−4^ M	99.83 ± 0.78	102.14 ± 0.57
10%	90%		
1.0 × 10^−4^ M	9.0 × 10^−4^ M	101.44 ± 0.66	101.55 ± 0.53

* Average of three measurements.

**Table 4:** Results obtained for the analysis of the tablet form using the fabricated membrane sensors at 25 °C.

Item	BRZ-SPE		BRZ-SCE
	Added (M)	Found (M) ± S.D. *	Found (M) ± S.D. *
Lexotanil^®^ tablets labeled to contain 3 mg BRZ per tablet (Batch No. M1139B01 and A506716)	10.0 × 10^−4^	10.1 × 10^−4^ ± 0.77	10.1 × 10^−4^ ± 0.72

* Average of five measurements.

**Table 5:** Statistical comparison for the determination of bromazepam in bulk powder using the fabricated sensors with a reported method.

Item	BRZ-SPE	BRZ-SCE	Reported Method [23] *
Mean ± S.D.	100.07 ± 1.06	100.42 ± 0.93	99.81 ± 0.59
RSD	1.06	0.93	0.59
Variance	1.12	0.86	0.35
n	5	5	5
Student’s *t*-test (2.30)	0.48	1.24	-
F-test (6.39)	3.25	2.50	-

* RP-HPLC- method using Nova Pak 5 microns C18 column as stationary phase, acetonitrile–water–triethylamine (700:300:4, by volumes) adjusted to pH 7.4 with orthophosphoric acid, as mobile phase, flow rate 1.0 mL/min, and UV detection at 240 nm.

## Data Availability

The datasets used and/or analyzed during the current study are available from the corresponding author on reasonable request.

## Supplementary Materials

The following supporting information can be downloaded at: https://www.mdpi.com/article/10.3390/molecules27217616/s1, Table S1: Original data for the calibration graphs for the BRZ electrodes using DOP as plasticizer at 25 °C; Table S2: Original data for the calibration graphs for the BRZ electrodes using DBS as plasticizer at 25 °C; Table S3: pH effect on the potential using two concentration levels of BRZ standard solutions; Table S4: Accuracy of the fabricated electrodes; Table S5: Robustness of the fabricated electrodes upon carrying out slight pH change (pH 6.2); Table S6: Ruggedness of the fabricated electrodes using Hanna digital ion analyzer; Figure S1: Calibration graphs for the BRZ electrodes using DOP as plasticizer at 25 °C; Figure S2: Calibration graphs for the BRZ electrodes using DBS as plasticizer at 25 °C.

## Sample Availability

Samples of the compounds are available from the authors.
