# Supplementary material for: A Comparative Study between Screen-Printed and Solid-Contact Electrodes for the Stability-Indicating Determination of Bromazepam
Source: Molecules. 2022 Nov 6;27(21):7616. doi: 10.3390/molecules27217616 (PMC9659083; doi:10.3390/molecules27217616)
Supplement: Supplementary file 1 [file molecules-27-07616-s001.zip › molecules-1943876-supplementary.pdf]

Supplementary data:

Table S1. Original data for the calibration graphs for the BRZ electrodes using DOP as plasticizer at 25 °C.

−log C	E (mV) SPE	E (mV) SCE
8	79.00	46.00
7	80.00	47.00
6	87.40	50.00
5	138.10	100.00
4	188.20	150.00
3	239.30	200.10
2	290.00	250.00

Table S2. Original data for the calibration graphs for the BRZ electrodes using DBS as plasticizer at 25 °C.

−log C	E (mV) SPE	E (mV) SCE
8	69.00	37.40
7	70.00	38.50
6	74.00	41.60
5	125.70	91.70
4	177.70	142.00
3	229.20	192.50
2	281.00	243.00

Table S3. pH effect on the potential using two concentration levels of BRZ standard solutions.

pH	10^{-3} M		10^{-4} M	
	SPE	SCE	SPE	SCE
	E (mV) ± SD *	E (mV) ± SD *	E (mV) ± SD *	E (mV) ± SD *
1.0	235.00 ± 0.85	194.00 ± 0.64	184.00 ± 1.16	150.00 ± 0.52
2.0	241.00 ± 0.63	200.00 ± 0.53	186.00 ± 0.81	151.00 ± 0.45
3.0	250.30 ± 0.76	211.90 ± 0.74	190.60 ± 1.31	153.8 ± 0.34
4.0	250.30 ± 1.01	211.90 ± 0.85	190.60 ± 0.77	153.8 ± 0.41
5.0	250.20 ± 0.45	211.80 ± 0.44	190.60 ± 0.99	153.8 ± 0.79
6.0	250.30 ± 0.50	211.90 ± 0.42	190.50 ± 0.70	153.70 ± 0.53
7.0	240.00 ± 0.66	201.00 ± 0.99	186.00 ± 1.42	151.00 ± 0.81
8.0	235.00 ± 0.94	195.00 ± 1.12	184.00 ± 0.54	150.00 ± 0.46
9.0	230.00 ± 0.81	190.00 ± 0.68	183.00 ± 1.51	149.00 ± 0.63
10.0	225.00 ± 0.42	185.00 ± 1.31	182.00 ± 0.72	148.00 ± 1.12

* Average of three measurements.

Table S4. Accuracy of the fabricated electrodes.

	Added concentration (M)	Found concentration (M)	Recovery%	Average recovery% ± SD
BRZ-SPE	10.00×10^{-3}	9.90×10^{-3}	99.00	100.07 ± 1.06
	10.00×10^{-4}	10.01×10^{-4}	100.10	
	10.00×10^{-5}	10.11×10^{-4}	101.10	
BRZ-SCE	10.00×10^{-3}	10.04×10^{-3}	100.40	100.42 ± 0.93
	10.00×10^{-4}	9.95×10^{-4}	99.50	
	10.00×10^{-5}	10.13×10^{-5}	101.30	

Table S5. Robustness of the fabricated electrodes upon carrying out slight pH change (pH 6.2).

	Added concentration (M)	Found concentration (M)	Recovery%	Average recovery% ± SD
BRZ-SPE	10.00×10^{-3}	10.11×10^{-3}	101.10	101.14 ± 1.13
	10.00×10^{-4}	10.23×10^{-4}	102.30	
	10.00×10^{-5}	10.00×10^{-4}	100.00	
BRZ-SCE	10.00×10^{-3}	10.08×10^{-3}	100.80	101.64 ± 0.84
	10.00×10^{-4}	10.23×10^{-4}	102.30	
	10.00×10^{-5}	10.16×10^{-5}	101.60	

Table S6. Ruggedness of the fabricated electrodes using Hanna digital ion analyzer.

	Added concentration (M)	Found concentration (M)	Recovery%	Average recovery% ± SD
BRZ-SPE	10.00×10^{-3}	10.08×10^{-3}	100.80	99.51 ± 1.28
	10.00×10^{-4}	9.82×10^{-4}	98.20	
	10.00×10^{-5}	9.95×10^{-4}	99.50	
BRZ-SCE	10.00×10^{-3}	10.01×10^{-3}	100.10	98.41 ± 1.66
	10.00×10^{-4}	9.84×10^{-4}	98.40	
	10.00×10^{-5}	9.68×10^{-5}	96.80	

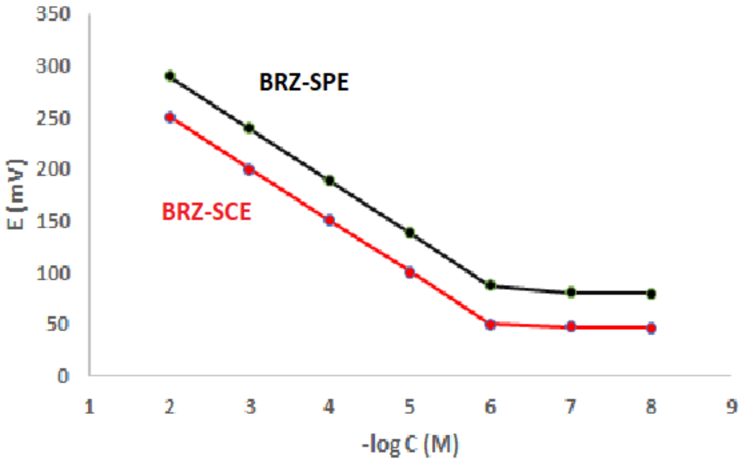

Figure S1. Calibration graphs for the BRZ electrodes using DOP as plasticizer at 25 °C.

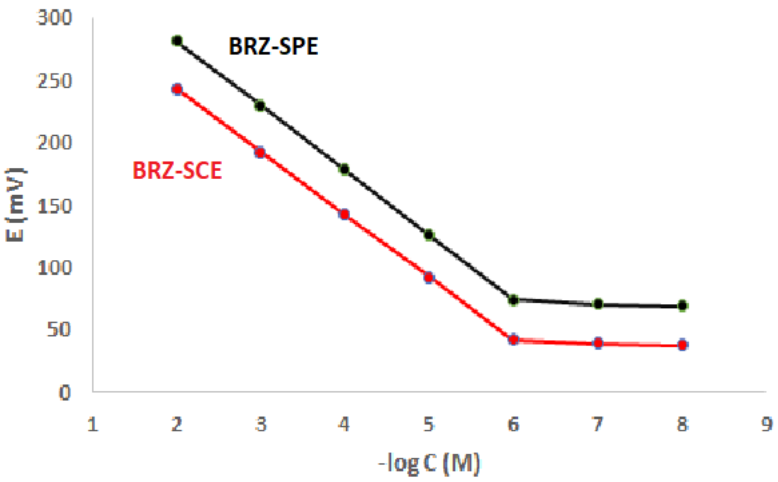

Figure S2. Calibration graphs for the BRZ electrodes using DBS as plasticizer at 25 °C.
